# A Novel Chemiluminescent Method for Efficient Evaluation of Heterogeneous Fenton Catalysts Using Cigarette Tar

**DOI:** 10.3390/toxics11010030

**Published:** 2022-12-29

**Authors:** Dabin Wang, Weisong Yu, Bin Jiang, Tao Zeng, Dean Song, Song Fang, Yizhi Zhang, Jiguang Zhang

**Affiliations:** 1 Laboratory of Quality & Safety Risk Assessment for Tobacco, Ministry of Agriculture, Tobacco Research Institute of Chinese Academy of Agricultural Sciences, Qingdao 266101, China; 2 Shandong Tobacco Company of China National Tobacco Company, Jinan 250101, China; 3 College of Environment, Zhejiang University of Technology, Hangzhou 310032, China

**Keywords:** chemiluminescence, heterogeneous fenton catalysis, cigarette tar, •OH detection, evaluation of catalytic capacity of catalysts

## Abstract

The evaluation of the catalytic capacity of catalysts is indispensable research, as catalytic capacity is a crucial factor to dictate the efficiency of heterogeneous Fenton catalysis. Herein, we obtained cigarette tar-methanol extracts (CTME) by applying methanol to cigarette tar and found that CTME could cause CL reactions with Fe^2+^/H_2_O_2_ systems in acidic, neutral, and alkaline media. The CL spectrum experiment indicated that the emission wavelengths of the CTME CL reaction with Fe^2+^/H_2_O_2_ systems were about 490 nm, 535 nm, and 590 nm. Quenching experiments confirmed that hydroxyl radicals (•OH) were responsible for the CL reaction for CTME. Then the CL property of CTME was applied in-situ to rapidly determine the amounts of •OH in tetrachloro-1,4-benzoquinone (TCBQ)/H_2_O_2_ system in acidic, neutral and alkaline media, and the CL intensities correlated the best (*R*^2^ = 0.99) with TCBQ concentrations. To demonstrate the utility of the CTME CL method, the catalytic capacity of different types and concentrations of catalysts in heterogeneous Fenton catalysis were examined. It was found that the order of CL intensities was consistent with the order of degradation efficiencies of Rhodamine B, indicating that this method could distinguish the catalytic capacity of catalysts. The CTME CL method could provide a convenient tool for the efficient evaluation of the catalytic capacity of catalysts in heterogeneous Fenton catalysis.

## 1. Introduction

Heterogeneous Fenton catalysis has become a major research focus in the area of wastewater treatment due to advantages over other advanced oxidation processes (AOPs) such as recyclability, wide pH response range, easy solid-liquid separation, and non-production iron sludge [1,2,3,4,5,6]. Catalytic capacity is the critical factor for dictating the efficiency of heterogeneous Fenton catalysis in the degradation of pollutants. Therefore, intensive attention has been paid to the synthesis of a wide variety of new catalysts to improve the catalytic capacity [7,8,9,10,11,12,13]. For example, Hu et al. broke through the traditional Fenton theory to synthesize a new type of catalyst with a dual-reaction center [14,15]. In practice, researchers usually synthesize a series of materials in different conditions to obtain, distinguish, and select a catalyst with the best catalytic capacity, which is important yet tedious work. Hydroxyl radical (•OH) plays a crucial role in the degradation of pollutants in heterogeneous Fenton catalysis, where the amount of •OH could be an indicator of the catalytic capacity of a catalyst. Therefore, it is feasible to evaluate the catalytic capacity of catalysts by rapid and in-situ detection of •OH in heterogeneous Fenton catalysis. The current detection methods for •OH mainly include electron spin resonance (ESR), ultraviolet-visible light (UV-vis) absorbance and fluorescence [16]. These methods usually need a capture probe to react with free radicals to form a detectable product, followed by solid-liquid separation and measurement, and could not carry out the rapid and in-situ detection of •OH in heterogeneous Fenton catalysis, which is not efficient for the evaluation of the catalytic capacity of catalysts.

Chemiluminescence (CL) is an optical phenomenon in which excited-state species generated through chemical reactions release energy (>45 kcal·mol^−1^) in the form of photons. CL is a well-suited method for the rapid detection of free radicals due to its fast detection speed and high sensitivity [17,18,19,20,21,22]. We have previously built a continuous flow CL method for rapid and dynamic monitoring of superoxide radicals in TiO_2_ photocatalysis by using the luminol CL system [23]. However, now there are currently no suitable known CL methods for rapid and in-situ detection of •OH in heterogeneous Fenton catalysis, which is largely because the pH value of the heterogeneous Fenton system is incompatible with the current CL reactions. Therefore, constructing a novel CL method suited to the heterogeneous Fenton system on the basis of the new principle of CL reaction would significantly contribute to efficiently evaluating the catalytic capacity of catalysts by rapid and in-situ detection of •OH.

Cigarette tar is the condensate product from incomplete combustion of tobacco under high-temperature and anoxic conditions. It has an abundance of various compounds, and though a fraction derives from the original composition of tobacco, most of the components are the products generated from cigarette combustion. To date, research has mainly focused on the hazardous components and their toxicological implications pertinent to cigarette tar [24,25]. In our previous study, we reported the CL property of tobacco extract [26]. For cigarette tar, there are probably some chemiluminophores directly transferred from tobacco. More importantly, however, is that an abundance of fused polycyclic compounds is produced in cigarette tar during combustion, which might be favorable for the chemical transformation of chemiluminophores with more aromaticity comparable to the current CL probes. This might eventually improve the luminous efficiency of cigarette tar in comparison with tobacco.

Based on the above analysis, our objective in this current study has been to develop a new method for efficiently evaluating the catalytic capacity of catalysts through rapid and in-situ detection of •OH. Therefore, we first explored the CL properties of cigarette tar-methanol extracts (CTME) and then examined the feasibility of the CTME CL method for the rapid and in-situ detection of •OH. Finally, the CTME CL method was demonstrated to be able to efficiently evaluate the catalytic capacity of catalysts in heterogeneous Fenton catalysis.

## 2. Materials and Methods

### 2.1. Chemicals and Materials

Tetrachloro-1,4-benzoquinone (TCBQ) was purchased from Aladdin Chemistry Co., Ltd. (Shanghai, China). FeCl_3_·6H_2_O, MnCl_2_·2H_2_O, CuCl_2_·2H_2_O, CoCl_2_·6H_2_O, Rhodamine B, and thiourea were purchased from Sinopharm Chemical Reagent Co., (Shanghai, China). A Cambridge filter was purchased from Borgwaldt (Hamburg, Germany). A Millipore membrane was obtained from ANPEL Laboratory Technologies Inc. (Shanghai, China). Ultrapure water (>18.2 MΩ) was used throughout all experiments.

### 2.2. Preparation of Cigarette-Tar-Methanol Extracts (CTME)

The cigarettes were made as follows: ripe fresh tobacco leaves were cured by three-stage-curing (yellowing, color fixing, and vein drying) procedures detailed in a previous report [26]. After curing, the leaves were cleaned through dust removal using a brush and then left to regain moisture for 10 h at room temperature. Then the tobacco leaves were cut into shreds after removing veins and made into cigarettes using a cigarette rolling machine. Each cigarette was about 70 mm in length, 27.5 mm in circumference, and 1.1 g in weight. In order to obtain the cigarette tar, 20 cigarettes were placed on a smoke machine (Borgwaldt, Germany), and the Cambridge filter was used to trap the particulate matter of the mainstream smoke. Then the filter was cut into strips, added to 40 mL of methanol, sonicated for 20 min, and filtered through 0.45 μm Millipore membrane to obtain the CTME (filtrate) for further experimentation. The final mass concentration of CTME used throughout the experiments was about 4.0 mg/mL.

### 2.3. Synthesis of Catalysts

Mesoporous MnFe_2_O_4_ and CoFe_2_O_4_ nanospheres were prepared by a modified hydrothermal method previously reported [27]. Typically, 1.35 g of FeCl_3_·6H_2_O and the corresponding transition metal salts (1.61 g MnCl_2_·2H_2_O and 2.37 g CoCl_2_·6H_2_O) with a molar ratio of 2:1 were dissolved in ethylene glycol (40 mL) containing 3.6 g of sodium acetate. The mixture was then covered and stirred vigorously on a magnetic stirrer for 30 min, and once a clear yellow solution was obtained, the solution was transferred to a Teflon-lined stainless-steel autoclave. Then, the autoclave was heated slowly to 200 °C and maintained for 8 h. The products were separated by applying an external magnetic field after the solution was cooled down to room temperature. The precipitate was washed several times with ethanol and dried under vacuum at 60 °C for 12 h. The FeOCl nanosheet was synthesized by heating FeCl_3_·6H_2_O at a rate of 10 °C·min^−1^ to 220 °C and annealing for 2 h, as previously reported [28].

### 2.4. CL Measurements

CL kinetic curves were recorded in batch experiments, which were conducted in a static system consisting of a glass cuvette and a BPCL Ultra-Weak Luminescence Analyzer (Institute of Biophysics, Chinese Academy of Sciences, Beijing, China). Briefly, for each CL reaction, 100 μL of CTME and involved reagents were respectively added into a glass cuvette. Then 100 μL of co-reaction reagent was injected using a microsyringe in the upper injection pore to trigger a CL reaction. For the measurement of the CL spectrum, a series of high-energy optical filters (440, 460, 475, 490, 505, 535, 555, 575, 590, and 605 nm) were utilized to screen the CL intensities of CTME CL systems, respectively.

### 2.5. Degradation of Rhodamine B

The degradation of Rhodamine B by different types and concentrations of catalysts in heterogeneous Fenton catalysis was conducted as follows. A total of 10 μL of Rhodamine B (5.0 mg/mL) was added into 5.0 mL of three kinds of catalysts (1.0 mg/mL ) or into different concentrations of FeOCl nanosheet (0.05, 0.08, 0.1, 0.2 and 0.5 mg/mL), followed by adding 500 μL of H_2_O_2_ (1.0 mol/L). After 10 min, 500 μL of ascorbic acid (0.5 mol/L) was added to the mixture to stop the reaction. Then the mixture was filtered through a 0.45 μm membrane, and the filtrate containing the residual Rhodamine B was measured on the UV-vis spectrophotometer.

## 3. Results

### 3.1. CL Property of CTME

**Figure 1:**
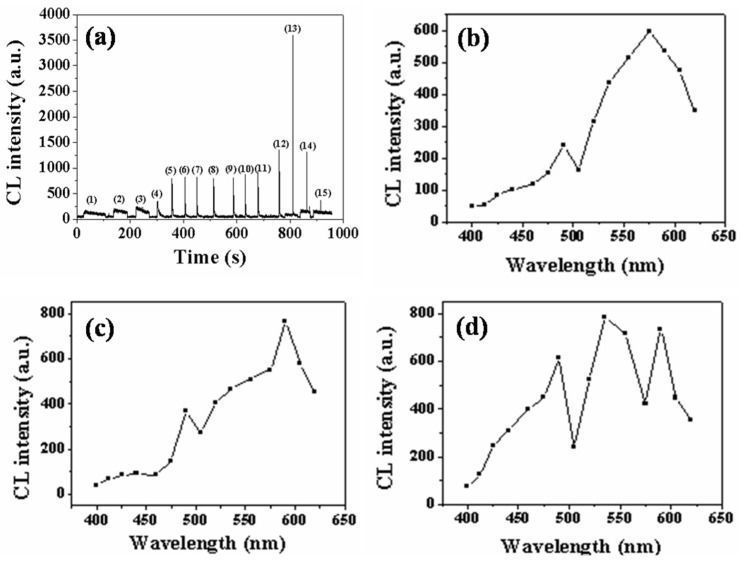
(**a**) CL kinetic curves of CTME (4.0 mg/mL) with FeSO_4_ (0.1 mM)/H_2_O_2_ (1.0 mM) systems under different pH conditions: H_2_SO_4_: (1) 1.0 mol/L, (2) 0.1 mol/L, (3) 0.01 mol/L, (4) 1 × 10^−3^ mol/L, (5) 1 × 10^−4^ mol/L, (6) 1 × 10^−5^ mol/L, (7) 1 × 10^−6^ mol/L, H_2_O: (8), NaOH: (9) 1 × 10^−6^ mol/L, (10) 1 × 10^−5^ mol/L, (11) 1 × 10^−4^ mol/L, (12) 1 × 10^−3^ mol/L, (13) 0.01 mol/L, (14) 0.1 mol/L, (15) 1.0 mol/L; CL spectrums of CTME (4.0 mg/mL) with FeSO_4_ (10.0 mM)/H_2_O_2_ (0.1 M) systems in (**b**) H_2_SO_4_ (0.1 mM), (**c**) H_2_O and (**d**) NaOH (0.01 M) solutions.

We have previously studied the CL behavior of tobacco-methanol extract (TME) with the Fe^2+^/H_2_O_2_ system [26]. Herein, the CL characteristics of CTME with the Fe^2+^/H_2_O_2_ system were also investigated. As shown in Figure 1a, the CL emissions of CTME with the Fe^2+^/H_2_O_2_ system were generated at different pH levels ranging from 0 to 14. Results indicated that CTME could undergo CL reactions with Fe^2+^/H_2_O_2_ system in acidic, neutral, and alkaline media as with the TME [26]. CTME exhibited slow CL reactions, which had almost a plateau of long-lasting weak emissions at pH ≤ 2, while there were fast CL reactions for TME, and the CL intensity reached the maximum at pH = 1 [26]. As pH increased, however, the CL intensity of CTME increased until pH 4 and remained stable from pH 4 to 10. From pH 11 to 14, the CL intensity of CTME escalated and then declined drastically. The maximum of CL intensity for CTME was at pH = 12, which was about three times higher than at pH 4 through 10. In contrast, the CL intensity for TME began to decrease at pH > 1 and increased to the maximum at pH = 9 once again [26]. Thereafter, the CL intensity declined [26]. The results show the different CL characteristics between CTME and TME, indicating that the chemiluminophores within CTME and TME were probably different in quantity and type. In addition, the luminescent efficiency of CTME and TME was also examined (Figure S1). The CL intensity of CTME was about two to three times greater than TME at the same mass concentration. This further implied that the process of combustion that generated cigarette tar from tobacco probably changed the chemiluminophores both in quantity and types, which lead to higher luminescent efficiency of CTME than TME.

To further examine the CL behavior of CTME with Fe^2+^/H_2_O_2_, CL spectrums of CTME-Fe^2+^/H_2_O_2_ were conducted in acidic, neutral, and alkaline media, respectively (Figure 1b–d). In an acidic medium with 0.1 mM of H_2_SO_4_ solution, there were two peaks, one centered at 490 nm and the other at 575 nm (Figure 1b). In H_2_O as the neutral medium, two maximum peaks appeared at about 490 nm and 590 nm (Figure 1c). The variation of wavelength shifted from 575 nm in an acidic medium to 590 nm in a neutral medium, which could probably be attributed to the change in pH value. In the 0.01 mol/L of NaOH solution representative of the alkaline medium, there was an additional peak relative to the two peaks at 490 nm and 590 nm that emerged at 535 nm (Figure 1d). Furthermore, the CL intensity of peak at 490 nm escalated as pH increased, while the CL intensity at 590 nm in neutral and alkaline media was almost identical but larger than that at 575 nm in the acidic medium. The CL intensities at 590 nm (or 575 nm in an acidic medium) were larger than those at 490 nm regardless of the pH value. CL intensity at 535 nm in an alkaline medium was higher than those both at 490 nm and 590 nm, which is most likely the reason that there was a maximum CL intensity in the 0.01 mol/L of NaOH solution. Overall, approximately three kinds of potential emitting species in CTME participated in CL reactions with Fe^2+^/H_2_O_2_, and the emission wavelength at 490 nm and 590 nm could undergo CL reactions regardless of the pH value for some of the species. This is intriguing given that the conventional CL reactions are usually restricted by pH value. Meanwhile, the emitting species corresponding to the emission wavelength at 535 nm tended to take place CL reaction in alkaline media (e.g., pH = 12), but not in acidic and neutral solutions.

### 3.2. •OH Detection

**Figure 2:**
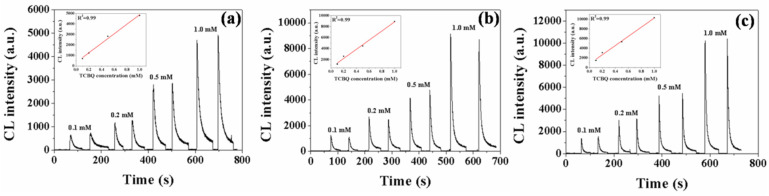
CL kinetic curves of CTME (4.0 mg/mL) with TCBQ (0.1, 0.2, 0.5, and 1.0 mM) and H_2_O_2_ (0.1 M) systems in (**a**) 1.0 mM H_2_SO_4_, (**b**) H_2_O and (**c**) 0.1 mM NaOH solutions. The inset graphs denote the linear relationship of CL intensity with TCBQ concentrations.

In our previous study, hydroxyl radical (•OH) was confirmed to be responsible for the CL reaction of TME. In our present study, a universal •OH scavenger thiourea was added to the CTME-Fe^2+^/H_2_O_2_ system to investigate the role of •OH in the CTME CL reaction (Figure S2). CL signals were completely inhibited by adding thiourea in acidic, neutral, and alkaline mediums, meaning that •OH played a crucial role in the CTME CL reaction. To verify the feasibility of the CTME CL method for determining •OH, a typical •OH-generating system, tetrachloro-1,4-benzoquinone (TCBQ)/H_2_O_2_, was adopted to conduct a CTME CL reaction [29]. The CL phenomenon of CTME was first investigated by mixing with TCBQ/H_2_O_2_ system in acidic, neutral, and alkaline media. CL emissions were all produced by CTME-TCBQ/H_2_O_2_ systems (Figure S3), indicating that •OH triggering CTME CL reactions in this system occurred in acidic, neutral, and alkaline solutions. Then the relationship between the CL intensity of CTME and the amount of •OH was conducted. Different •OH amounts were indirectly made by changing the TCBQ concentration due to its short lifetime. As shown in Figure 2, the CL intensity of CTME exhibited a linear increase with TCBQ concentrations (*R*^2^ = 0.99) in acidic, neutral, and alkaline media, confirming that the CL intensity of CTME was •OH concentration-dependent in TCBQ/H_2_O_2_ systems. These results also confirmed that the CTME CL method could achieve the rapid and in-situ detection of •OH in a semi-quantitative way.

### 3.3. Evaluation of the Catalytic Capacity of Catalysts

**Figure 3:**
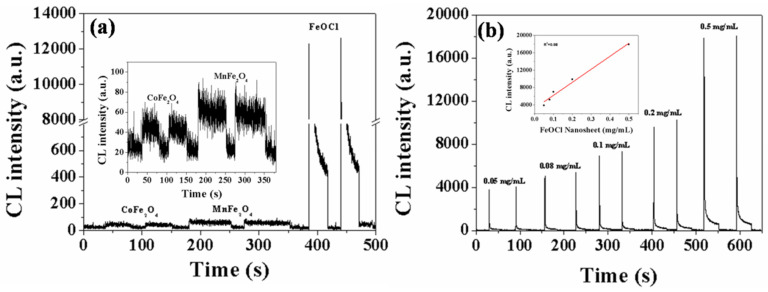
(**a**) CL kinetic curves of CTME (4.0 mg/mL) with three catalysts (1.0 mg/mL)/H_2_O_2_ (0.1 mol/L) systems (The inset graph denotes the enlarged CL kinetic curves of CTME with CoFe_2_O_4_ and MnFe_2_O_4_), and (**b**) with different concentrations of FeOCl/H_2_O_2_ (0.1 mol/L) systems (The inset graph denotes the linear relationship of CL intensity with FeOCl concentrations).

The heterogeneous Fenton catalytic reaction has been a research hotspot for water treatment technology due to its advantages in comparison with other AOPs [6]. Now researchers are keen on synthesizing various catalysts for heterogeneous Fenton catalysis, and thus the ability to distinguish catalytic capacity is indispensable. The catalytic capacity of these synthesized catalysts is highly dependent on •OH production. Herein, we attempted to evaluate the catalytic capacity of the three different catalysts (FeOCl, CoFe_2_O_4,_ and MnFe_2_O_4_) under the same experimental conditions, and then the same catalysts (FeOCl) with different concentrations by determining the amount of •OH in-situ and rapidly with the CTME CL method. Of the three catalysts shown in Figure 3a, the highest CL intensity was derived from FeOCl, which was much more intense than CoFe_2_O_4_ and MnFe_2_O_4_, but the CL intensity of MnFe_2_O_4_ is only slightly larger than that of CoFe_2_O_4_. For individual FeOCl (Figure 3b), the CL intensity of CTME increased as the concentration of FeOCl increased, and there was a good correlation between them (*R*^2^ = 0.98). In addition, degradation efficiencies of Rhodamine B with different types and concentrations of catalysts were also performed under the same conditions (Figure S4). The order of Rhodamine B degradation efficiency for three catalysts was FeOCl > MnFe_2_O_4_ > CoFe_2_O_4_ (Figure S4a), in accordance with the CL intensity in Figure 3a. Figure S4b showed that the degradation efficiency of Rhodamine B increased with the FeOCl concentration, which was also consistent with the CL intensity in Figure 3b. The combined results in Figure 3 and Figure S4 strongly confirmed that the CTME CL method could efficiently evaluate the catalytic capacity of catalysts in heterogeneous Fenton catalysis.

## 4. Conclusions

In this work, the CL property of CTME was examined with •OH at different pH values, and subsequently achieved the rapid and in-situ detection of •OH in a semi-quantitative way in acidic, neutral, and alkaline media. Then the CTME CL method was successfully used to evaluate the catalytic capacity of catalysts in heterogeneous Fenton catalysis. Given that numerous catalysts have been synthesized for heterogeneous Fenton catalysis, the CTME CL method provides a convenient tool for the efficient evaluation of the catalytic capacity. In addition, the chemiluminophores within CTME are also intriguing and worthy of further research.

## Data Availability

All data analyzed during this study are included in this published article.

## Supplementary Materials

The following supporting information can be downloaded at: https://www.mdpi.com/article/10.3390/toxics11010030/s1, Figure S1: CL kinetic curves of CTME and TME (3.5 mg/mL) with FeSO_4_ (0.1 mM)/H_2_O_2_ (1.0 mM) in a neutral medium; Figure S2: CL intensity of CTME (4.0 mg/mL) with FeSO_4_ (0.1 mM)/H_2_O_2_ (1.0 mM) systems in H_2_SO_4_ (0.1 mM), H_2_O and NaOH (0.01 M) solutions before and after the addition of thiourea; Figure S3: CL emissions of CTME (4.0 mg/mL) with TCBQ (1 mM)/H_2_O_2_ (0.01 M) systems in (a) H_2_SO_4_ (1.0 mM), (b) H_2_O and (c) NaOH (0.1 mM) solutions; Figure S4: Degradation efficiency of Rhodamine B with (a) three catalysts (0.1 mg/mL)/H_2_O_2_ (0.1 mol/L) systems, and (b) different concentrations of FeOCl/H_2_O_2_ (0.1 mol/L).
