# Supplementary material for: A Novel Chemiluminescent Method for Efficient Evaluation of Heterogeneous Fenton Catalysts Using Cigarette Tar
Source: Toxics. 2022 Dec 29;11(1):30. doi: 10.3390/toxics11010030 (PMC9866030; doi:10.3390/toxics11010030)
Supplement: Supplementary file 1 [file toxics-11-00030-s001.zip › toxics-2044042-supplementary.pdf]

toxics

MDPI

Supplementary Materials: A Novel Chemiluminescent Method for Efficient Evaluation of Heterogeneous Fenton Catalysts Using Cigarette Tar

Dabin Wang, Weisong Yu, Bin Jiang, Tao Zeng, Dean Song, Song Fang, Yizhi Zhang and Jiguang Zhang

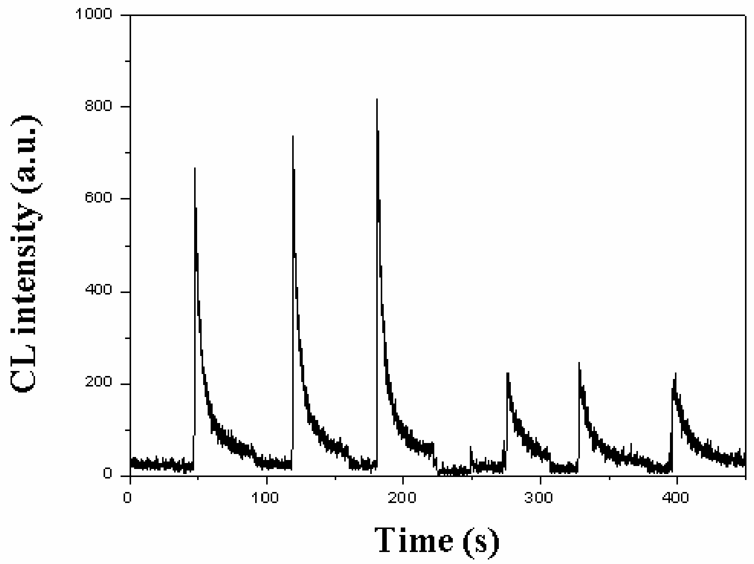

Figure S1. CL kinetic curves of CTME and TME (3.5 mg/mL) with $FeSO_4$ (0.1 mM)/H_2O_2 (1.0 mM) in neutral medium.

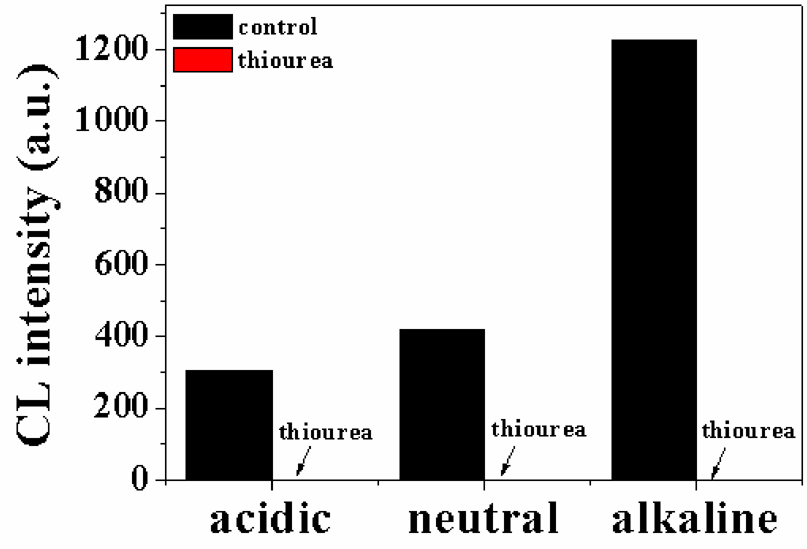

Figure S2. CL intensity of CTME (4.0 mg/mL) with $FeSO_4$ (0.1 mM)/H_2O_2 (1.0 mM) systems in H_2SO_4 (0.1 mM), H_2O and NaOH (0.01 M) solutions before and after the addition of thiourea.

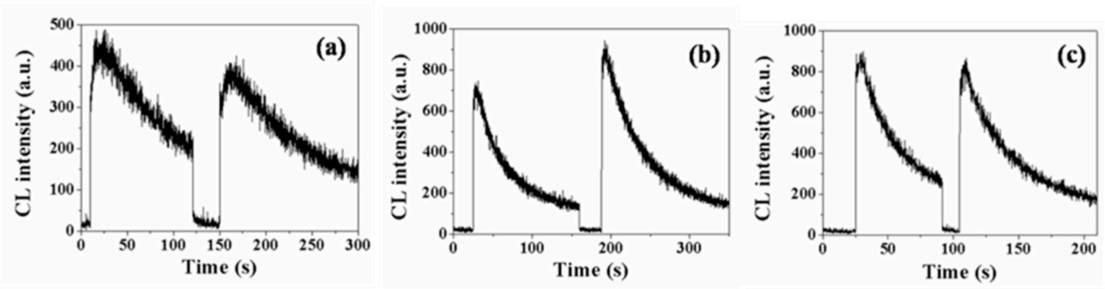

Figure S3. CL emissions of CTME (4.0 mg/mL) with TCBQ (1 mM)/H_2O_2 (0.01 M) systems in **(a)** H_2SO_4 (1.0 mM), **(b)** H_2O and **(c)** NaOH (0.1 mM) solutions.

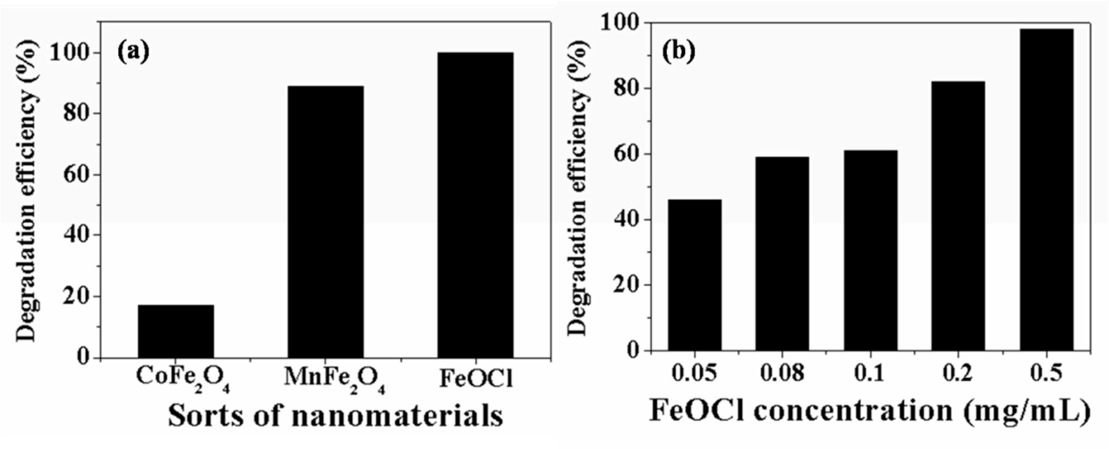

Figure S4. Degradation efficiency of Rhodamine B with **(a)** three catalysts (0.1 mg/mL)/H_2O_2 (0.1 mol/L) systems, and **(b)** different concentrations of FeOCl/H_2O_2 (0.1 mol/L).
